# Supplementary material for: Geographical origin determines responses to salinity of Mediterranean caddisflies
Source: PLoS One. 2020 Jan 13;15(1):e0220275. doi: 10.1371/journal.pone.0220275 (PMC6957138; doi:10.1371/journal.pone.0220275)
Supplement: S1 Table — (DOCX) [file pone.0220275.s001.docx]

**Table S1:** **Repeatability and responses of each behavioral trait.** Intraclass Correlation Coefficient (ICC) as indicator of repeatability, and ANOVA with generalized linear model (GLMs) for behavioral responses of *Smicridea annulicornis.* Geographical origin (basins Choapa, Maipo and Maule), conductivity levels (low, medium, high), age and body length were used as predictor factors. Statistical significance: * P < 0.05, ** P < 0.01, *** P < 0.001.

| **Trait** | **Factor** | **F-value _(df)_** |
| --- | --- | --- |
| **Swimming** | Age | 5.27 _(1,203)_* |
| ICC  0.04 ± 0.01 (SE) | Body length | 4.07 _(1,202)_* |
|  | Conductivity | 1.93 _(2,200)_ |
|  | Basin | 14.9 _(2,198)_*** |
|  | Conductivity × basin | 1.95 _(4,194)_ |
| **Pushing-up** | Age | 4.99 _(1,203)_* |
| ICC  0.02 ± 0.006 (SE) | Body length | 0.06 _(1,202)_ |
|  | Conductivity | 0.36 _(2,200)_ |
|  | Basin | 23.7 _(2,198)_*** |
|  | Conductivity × basin | 0.57 _(4,194)_ |
| **Walking** | Age | 0.26 _(1,203)_ |
| ICC  0.15 ± 0.03 (SE) | Body length | 0.07 _(1,202)_ |
|  | Conductivity | 0.43 _(2,200)_ |
|  | Basin | 2.29 _(2,198)_ |
|  | Conductivity × basin | 0.82 _(4,194)_ |
| **Sheltering** | Age | 11.9 _(1,203)_*** |
| ICC  0.13 ± 0.03 (SE) | Body length | 23.8 _(1,202)_*** |
|  | Conductivity | 1.21 _(2,200)_ |
|  | Basin | 2.12 _(2,198)_ |
|  | Conductivity × basin | 0.58 _(4,194)_ |
