# Supplementary material for: Geographical origin determines responses to salinity of Mediterranean caddisflies
Source: PLoS One. 2020 Jan 13;15(1):e0220275. doi: 10.1371/journal.pone.0220275 (PMC6957138; doi:10.1371/journal.pone.0220275)
Supplement: Attachment — Submitted filename: Response to Reviewers.docx [file pone.0220275.s002.docx]

PONE-D-19-19484
Geographical origin determines responses to salinity of Mediterranean caddisflies
PLOS ONE

Response to reviewers (point-by-point)

**Journal Requirements:**

When submitting your revision, we need you to address these additional requirements.

Please ensure that your manuscript meets PLOS ONE's style requirements, including those for file naming. Response: Checked

1. In your Methods section, please provide additional location information of the study areas, including geographic coordinates for the data set if available.

Response: Done as requested. L 79-80

2. In your Methods section, please provide additional information regarding the permits you obtained for the work. Please ensure you have included the full name of the authority that approved the field site access and, if no permits were required, a brief statement explaining why.

Response: No permits are required, according to national regulations. We included this information in the text. L 80-82

3. We note that you have stated that you will provide repository information for your data at acceptance. Should your manuscript be accepted for publication, we will hold it until you provide the relevant accession numbers or DOIs necessary to access your data. If you wish to make changes to your Data Availability statement, please describe these changes in your cover letter and we will update your Data Availability statement to reflect the information you provide.

Response: We uploaded the data to the dryad repository. We included this information in the cover letter as requested.

**Review Comments to the Author**

**Reviewer #1:**

According to the instructions to authors, the authors either need to place the data in a public repository, where URL, accession numbers or DOIs can be cited, or can be contained in Supporting Information files. The current statement says that the data will be available upon request.

Response: We uploaded the data to the dryad repository. We included this information in the cover letter as requested.

The paper needs a little editing, but, per instructions, I did not note specific edits, except that in the second sentence of the Introduction, the verbs should be "disrupts" and "threatens".
Response: Corrected. L 4-5

The authors might consider discussing how a species might adapt to differences in salinity across its range. At higher taxonomic levels, adaptation to different salinities can involve small mutations to the genes for the ion transporter genes that affect influx and efflux rates. In anadromous or catadromous species, such as salmonids and eels, adaptation may involve up-regulation or down-regulation of these ion transporters or of entire ionocytes.
Response: Corrected. In the 2^nd^ paragraph of the Discussion section we now talk about rapid evolution and mechanisms of ion regulation in aquatic organisms, including macroinvertebrates. L 210-227

**Reviewer #2:**

One factor that is only lightly addressed is this species in this region exhibits significant genetic differentiation among basins (Sabando et al. 2011) – suggesting either local adaptation with little or no gene exchange, or possibly some closely related species or subspecies that are unrecognized and responding to a strong habitat template.
Response: We extend our discussion about the empirical evidence of genetic structure in our study species on the study sites, and also include the reviewer’s idea of unrecognized species or subspecies. L 237-242

Specific comments;

I assume the experiments were conducted at 20 C, same as the acclimation temperature.

Response: Yes. This information was included in the revised version. L 102-104

Survival time – Table 1 indicates that basin matters, and that the effect of conductivity was not significant (p=0.08), but the results present a bunch of antidotal assessments of conductivity effects. Table 1 results and the Survival results need to be in agreement – at least justify the details by calling the factorial result “nearly significant” and not “marginal”.
Response: We corrected the text following the reviewer's suggestions. L 148

In the behavioral tests, why do you think the BC x Basin was significant? Why would behavioral responses differ between basins within a species?

Response: Done. We added a phrase in which we –with caution-- mention possible causes. L 232-235

In the behavioral tests, do you believe behavioral responses are in some what adaptive with regard to salinity?

Response: Our results do not allow inferring that behavioral responses are adaptive respect to salinity. We guess that the previous response covers this aspect too.

The results presented here make one question standard bioassay results – where was the original test population from? Have laboratory conditions changed the test organisms?

Response: Corrected. We commented on the limitation of lab experimentation. 250-253
